# Subtle Cognitive Impairments and Psychological Complaints in Patients With Prolactinoma Despite Biochemical Control

**DOI:** 10.1210/clinem/dgaf355

**Published:** 2025-06-13

**Authors:** Victoria R van Trigt, Cornelie D Andela, Leontine E H Bakker, Steffanie C M Brama, Lotte E Schmidt, Florian M Sneekes, Margot W Zeelenberg, Sasja D Huisman, Stephanie E E C Bauduin, Olaf M Dekkers, Marco J T Verstegen, Wouter R van Furth, Iris C M Pelsma, Nienke R Biermasz

**Affiliations:** Department of Medicine, Division of Endocrinology, and Center for Endocrine Tumors Leiden, Leiden University Medical Center, Leiden, 2333 ZA, the Netherlands; Department of Medicine, Division of Endocrinology, and Center for Endocrine Tumors Leiden, Leiden University Medical Center, Leiden, 2333 ZA, the Netherlands; Department of Rehabilitation Medicine, Leiden University Medical Center, Leiden, 2333 ZA, the Netherlands; Department of Medicine, Division of Endocrinology, and Center for Endocrine Tumors Leiden, Leiden University Medical Center, Leiden, 2333 ZA, the Netherlands; Department of Medicine, Division of Endocrinology, and Center for Endocrine Tumors Leiden, Leiden University Medical Center, Leiden, 2333 ZA, the Netherlands; Department of Medicine, Division of Endocrinology, and Center for Endocrine Tumors Leiden, Leiden University Medical Center, Leiden, 2333 ZA, the Netherlands; Department of Medicine, Division of Endocrinology, and Center for Endocrine Tumors Leiden, Leiden University Medical Center, Leiden, 2333 ZA, the Netherlands; Department of Medicine, Division of Endocrinology, and Center for Endocrine Tumors Leiden, Leiden University Medical Center, Leiden, 2333 ZA, the Netherlands; Department of Medicine, Division of Endocrinology, and Center for Endocrine Tumors Leiden, Leiden University Medical Center, Leiden, 2333 ZA, the Netherlands; Department of Psychiatry, Leiden University Medical Center, Leiden, 2333 ZA, the Netherlands; Department of Medicine, Division of Endocrinology, and Center for Endocrine Tumors Leiden, Leiden University Medical Center, Leiden, 2333 ZA, the Netherlands; Department of Neurosurgery, Leiden University Medical Center, University Neurosurgical Center Holland, Leiden, 2333 ZA, the Netherlands; Department of Neurosurgery, Leiden University Medical Center, University Neurosurgical Center Holland, Leiden, 2333 ZA, the Netherlands; Department of Medicine, Division of Endocrinology, and Center for Endocrine Tumors Leiden, Leiden University Medical Center, Leiden, 2333 ZA, the Netherlands; Department of Medicine, Division of Endocrinology, and Center for Endocrine Tumors Leiden, Leiden University Medical Center, Leiden, 2333 ZA, the Netherlands

**Keywords:** prolactinoma, cognitive functioning, psychological complaints, maladaptive personality traits, dopamine agonists, transsphenoidal surgery

## Abstract

**Purpose:**

To assess cognitive functioning and psychological complaints in patients with biochemically controlled prolactinoma.

**Methods:**

Cross-sectional study comparing otherwise healthy patients treated for prolactinoma to age-, gender-, and education level-matched controls. The cognitive assessment included 8 tests assessing memory, verbal fluency, processing speed, selective attention, and executive functioning. Additionally, patients completed 7 validated questionnaires on psychological complaints. Generalized estimating equations were performed. *P* values <.050 were considered significant.

**Results:**

Sixty patients (controlled on dopamine agonists, n = 30; in surgical remission, n = 30), among whom 41 (68.3%) females, aged 42.3 ± 11.7 years, were compared to 60 matched controls. Patients scored significantly lower on assessments for verbal memory (fewer words on Verbal Learning Test of Rey: β = −1.8; 95% CI, −2.7 to −1.0), selective attention (fewer correct digits on Digit Deletion Test [β = −8.8; 95% CI, −16.2 to −0.2], longer time on Trial Making Test A [β = 5.2; 95% CI, 3.2 to 7.2]), and processing speed (fewer correct substitutions on Digit-Symbol Substitution Test: β = –4.2; 95% CI, −8.2 to −0.2). Furthermore, patients reported higher degrees of apathy (Apathy Scale: β = 2.4; 95% CI, 0.6 to 4.1), irritability (Irritability Scale: β = 2.2; 95% CI, 0.3 to 4.1), fatigue (Fatigue Severity Scale: β = 6.7; 95% CI, 2.7 to 10.8), and anxiety and depressive symptoms (Hospital Anxiety and Depression Scale, anxiety: β = 1.1; 95% CI, 0.1 to 2.1; depression: β = 1.7; 95% CI, 0.8 to 2.7). Tests assessing executive functioning and task switching were comparable in patients and matched controls.

**Conclusion:**

Compared to matched controls, patients with biochemically controlled prolactinoma showed subtle cognitive impairments (ie, memory, attention, and processing speed) and reported more psychological complaints. Physicians should be aware of these impairments and address them adequately.

Patients treated for prolactinoma suffer from various physical symptoms, including headaches, galactorrhea, and hypogonadism, leading to subfertility and menstrual cycle disturbances in females (1). Besides physical symptoms and complaints, patients frequently report a cognitive and psychological burden (2).

Treatment is predominately pharmacological with dopamine agonists (DA), although the most recent consensus statement suggests consideration of transsphenoidal surgery (TSS) for noninvasive prolactinomas (1). Both interventions are effective in achieving normoprolactinemia (3), yet their effect on cognitive and psychological functioning remains largely unknown. A recent literature review including 18 mostly cross-sectional studies with high risks of bias, indicated improvement, but not always normalization of mental wellbeing after biochemical normalization (4). Song et al studied cognitive functioning in patients with prolactinoma and demonstrated that patients in surgical remission (n = 20) showed better response activation and inhibition compared to patients with active prolactinoma (n = 20) (5). Moreover, amelioration of cognitive functions was observed in a heterogeneous group of patients operated for pituitary adenomas including 12 patients with prolactinoma (6). Furthermore, Montalvo et al demonstrated that cabergoline use was associated with improvement of cognitive functioning (ie, processing speed, working memory, visual learning, and problem solving) in a small group of patients with prolactinoma (n = 7) (7). A main limitation of these studies is the lack of control groups (accounting for confounders) and comparison between treatment modalities.

Considering the self-reported burden due to cognitive and psychological complaints before and after treatment, insight into cognitive functioning and psychological complaints after surgery or medical treatment would be valuable. Therefore, this cross-sectional study reports on cognitive functioning and psychological complaints in patients with prolactinoma treated medically or surgically who do not have overt psychopathological comorbidity, in comparison to matched healthy controls. Based on clinical experience and previous findings, treated patients were hypothesized to demonstrate remaining impairments in cognitive functioning and psychological complaints, and that these impairments would positively correlate with prolactin levels at diagnosis, hypopituitarism, and symptoms of anxiety and depression, which improve with longer durations of biochemical control/remission. Furthermore, patients in surgical remission were hypothesized to have less impairments than DA-controlled patients, as DAs may cause cognitive and psychological side effects.

## Methods

### Study Design and Participants

This cross-sectional study compared cognitive functioning and patient-reported psychological complaints in patients with prolactinoma (aged 18-70 years) without any confirmed psychiatric comorbidity to matched controls. The study was approved by the Science Committee (W2020.020), and all participants gave digital informed consent.

Two patient groups were studied: normoprolactinemic patients with prolactinoma (prolactin below upper limit of normal) (1) controlled on a stable DA dose for ≥6 weeks, or (2) in surgical remission for ≥6 months after TSS. Prolactinoma diagnosis was based on the combination of symptomatic hyperprolactinemia, a pituitary mass on magnetic resonance imaging (MRI), and exclusion of nontumorous causes of hyperprolactinemia. Healthy controls were matched 1:1 to the patients based on age (<10-year age difference), gender, and education level (ie, low, medium, and high, based on the guidelines of Statistics Netherlands (8)) at the time of the cognitive assessment. Controls were recruited either through referrals by patients or via advertisements if the patients could not provide a control. All participants were compensated for travel costs and controls who were recruited via advertisements received a 20 euro gift voucher.

Exclusion criteria were current pregnancy, current or past drug or alcohol abuse, use of medication known to reduce cognitive functioning (eg, opiates, benzodiazepines, antihistamines), previous (pituitary) radiotherapy, major comorbidity (eg, severe kidney, liver, cardiac, systemic inflammatory disease, malignancy), neurological pathology (eg, cerebrovascular accident, cerebral trauma, dementia, epilepsy), (history of) any psychiatric condition (eg, obsessive compulsive disorder, anxiety disorder, depression, attention deficit disorder, attention deficit hyperactivity disorder, burn-out). Strict exclusion of all psychopathology was performed to avoid bias. Healthy controls had no physical or psychiatric conditions, no medication use (contraceptives were accepted), and no current or past alcohol or drug abuse.

### Prolactinoma Treatment

Patients were treated at the outpatient clinic of the Leiden University Medical Center, a tertiary referral center for pituitary care, according to international guidelines, following a previously described Value-Based Health Care pathway (1, 9, 10). Most DA-treated patients were on cabergoline, which was up-titrated if needed, aiming at the minimal dose to maintain normoprolactinemia (1). Surgically treated patients underwent endoscopic TSS in a dedicated care protocol described previously (11). The surgical indication was typically DA intolerance. Hypopituitarism was assessed dynamically on clinical indication and substituted following international guidelines (12).

### Study Procedures

All participants completed an online set of validated questionnaires, ie, patient-reported outcomes measures (PROMs), in the week before the cognitive assessment. All participants underwent an extensive cognitive assessment performed by 1 of 5 trained researchers (S.C.M.B., L.E.S., F.M.S., M.W.Z., V.R.v.T.) following a standardized protocol, as shown in Supplementary File 1 (13, 14). All assessments were conducted under quiet conditions in a separate hospital room. Patients and their matched controls were evaluated at the same time of day to account for circadian fluctuations in cognitive functions. Two researchers independently scored cognitive test performance. Disagreements were resolved by discussion. In case of persisting disagreement, the majority vote was selected through consultation of a third researcher. The second and third correctors were blinded for the type of participant.

### Cognitive Assessment

The cognitive assessment consisted of 8 tests (outlined in the following section), and lasted 60 to 75 minutes in total. Detailed information on the tests and their scoring is provided in Supplementary Table S1 (13, 14).

#### The Verbal Learning Test of Rey

Fifteen unrelated words were shown and read to the participant, to be reproduced by the participant (15). The test consists of 5 rounds of reproduction: immediate reproduction (first 4 rounds) and a delayed reproduction (after 20 minutes). This study reports reproduction round 1, 2, 4, and the delayed reproduction—providing the most relevant information. More reproduced words indicate better verbal memory (scale 0-15).

#### Wechsler Adult Intelligence Scale Digit Span Task

A series of digits are read aloud by the investigator to be reproduced by the participant in 3 rounds: digit span forward (identical order), digit span backward (reversed order), and sequencing (ascending order). Higher scores per round indicate better verbal memory and working memory (scale 0-16 per round) (16).

#### Rey Complex Figure Test

Participants copy a complex figure and reproduce the figure from memory after 3 minutes (immediate recall) and 30 minutes (delayed recall) (17). Higher scores indicate better visuospatial memory (scale 0-36).

#### Wechsler Adult Intelligence Scale Digit-Symbol Substitution Test

Participants substitute as many numbers with indicated symbols as possible within 2 minutes (18). More correctly substituted numbers indicate better selective attention and processing speed (scale 0-135).

#### Digit deletion test

Participants are presented with a form containing 800 digits (numbers 1-9), in which they cross out numbers 3 and 7 diagonally and underline number 4. The number of correctly edited, incorrectly edited, and missed numbers within 3 minutes are marked (19). More correctly edited numbers indicate better selective attention and processing speed (scale 0-240).

#### Trail Making Test

In the Trail Making Test (TMT)-A, participants connect circles with numbers (numbers 1-25) in ascending order. In TMT-B, participants connect numbers (number 1-13) and letters (letter A-L) in alternating order (20). Shorter duration to perform the task and fewer mistakes indicate better selective attention (TMT-A) or cognitive flexibility (TMT-B).

#### Delis-Kaplan Executive Function System Tower Test

Participants replicate 9 towers by stacking 5 differently sized disks onto 3 wooden pegs, never stacking larger disks on top of smaller disks, and only moving 1 disk at a time (21). A total performance score, average time to first step, time-per-step-ratio, step-accuracy-ratio, and rule-violations-per-item-ratio are noted. Higher scores indicate better executive functioning (scaled scores range 0-10).

#### FAS

Participants produce as many words as possible in 1 minute beginning with an F, A, and S, respectively (22). More correct, and fewer incorrect words and fewer repetitions indicate better executive functioning and verbal fluency (no maximum score).

### Patient-reported Outcomes Measures

Participants completed an online set of either 6 or 7 validated PROMS (duration approximately 60 minutes). The PROMs are summarized below. Detailed explanations and scoring are provided in Supplementary Table S2 (13, 14).

The first questionnaire, Leiden Bothers and Needs Pituitary (LBNQ-Pituitary) was only completed by patients to assess clinical characteristics, including relevant questions concerning psychological and cognitive functioning. This questionnaire measures pituitary-disease burden (Bothers) and the Needs for attention for these symptoms by the treating physician. It consists of 5 separate subscales and a total score for Bothers and Needs, respectively (33 items, scale 0-100 with higher scores indicating higher disease burden) (23).

The following 6 questionnaires were completed by all participants. The Apathy Scale and Irritability Scale measure various aspects of apathy and irritability, respectively (14 items, scale 0-42 with higher scores indicating greater apathy or irritability, respectively) (24, 25). Scores ≥14 on either instrument indicate participants being apathic or irritable, respectively. The *Fatigue Severity Scale* measures the effect of fatigue on health-related quality of life (9 items; scale 9-63, with higher scores indicating more fatigue) (26). The Hospital Anxiety and Depression Scale (HADS) describes the severity of depressive symptoms and anxiety (14 items, total score scale 0-21) (27, 28), with a separate subscale for depressive symptoms and anxiety (subscores ≥8: clinically relevant depressive symptoms or anxiety). The Irrational Beliefs Inventory-50 measures the extent of irrational beliefs including 5 subscales (50 items; scale 0-250, with higher scores indicating stronger irrational beliefs) (29). The Dutch Clinical Personality Questionnaire (DCPQ) measures 5 personality traits: Negativism, Shyness, Extraversion, Narcissism, and Severe Psychopathology (120 items; scale 0-40 per trait, with higher scores indicating a higher chance of having the trait) (30).

### Study Parameters

Patient demographics, biochemical analyses, and radiologic examinations were derived from our prospective database (31). Demographic information from controls and additional information from all participants were acquired during a short interview as part of the cognitive assessment protocol (Supplementary File 1 (13, 14)). Adenoma remnants were subdivided by current size: micro < 10 mm, macro 10 to 40 mm, and giant > 40 mm. Disease duration was defined as the time from diagnosis to the cognitive assessment (months). Duration of biochemical control/remission was defined as duration from sustained normoprolactinemia to the cognitive assessment (months). For this study, subtle cognitive impairment was defined as significantly worse scores compared to healthy controls, without deviations >2.0 SD from the control group's mean.

### Data Analysis

Data were analyzed using IBM SPSS statistics 29 (IBM Corp. Armonk, NY, USA) and reported as mean ± SD or median (interquartile range) depending on the normality of the data distribution for continuous variables, or frequency (percentage) for dichotomous variables. For comparison of baseline characteristics between patients and controls, an independent *t*-test was used for continuous variables and a χ^2^ test for categorical data.

The primary analysis compared the outcomes of the cognitive assessment and PROMs of treated patients with (1:1) matched controls using generalized estimating equations accounting for matching without correction for additional factors as baseline characteristics were not statistically nor clinically relevantly different. *Z*-scores and 95% CI for cognitive test results were calculated based on the patients’ matched controls to account for differences in age, gender, education, and time of testing.

The secondary analyses compared (1) the outcomes of the cognitive assessment and PROMs of patients controlled on DA with patients in surgical remission using generalized linear models of *Z*-scores; 95% CI and β were reported. (2) Subsequently, factors of influence on the cognitive assessment were estimated by multilinear regression analyses using *Z*-scores for the outcomes of the cognitive assessment as dependent factors, and serum prolactin at diagnosis, hypopituitarism (yes/no), duration of biochemical control/remission, and total HADS score as predictive factors. Appropriateness assumptions were evaluated using scatter plots, probability-probability plots, residual statistics, and Cooks tests. Baseline prolactin and duration of biochemical control/remission were log-transformed because they were not normally distributed. Regression analyses were only performed for outcomes of the cognitive assessment that differed between patients and controls in the primary analysis to limit the number of analyses. Again, 95% CI and standardized β were reported.

A *P* value ≤.050 was considered significant because of the exploratory nature of the study and the underlying association of the endpoints, as the tests cover partially overlapping cognitive domains. To avoid overcorrection, it was more appropriate to present outcomes with interval estimations, evaluating general patterns, than to merely perform hypothesis testing with correction for multiplicity (32).

## Results

### Full Cohort

#### Inclusion of patients and clinical characteristics

**Figure 1.**
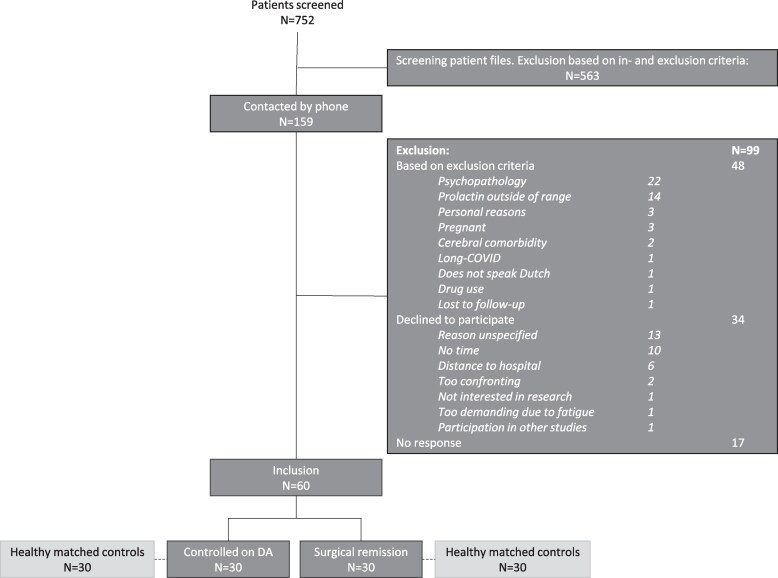
Flowchart of patient inclusion. Abbreviation: DA, dopamine agonist.

The flowchart of patient inclusion is depicted in Fig. 1. Eligible patients were invited to participate by phone or email (n = 159). In total, 142 patients were successfully contacted, of whom 48 were excluded based on in- and exclusion criteria (among which 22 because of psychopathology) and 34 patients declined to participate, primarily citing lack of time or travel distance as reasons (unspecified reason, n = 13).

Thus, 60 patients were included (41 females [68.3%]), mean age 42.3 ± 11.7 years, whose characteristics are summarized in Table 1. The patients’ education levels were classified as either high (n = 37, 61.7%), medium (n = 19, 31.7%), or low (n = 4, 6.7%). Of these, 30 patients were controlled on DA (17 females [56.7%]) and 30 patients were in surgical remission (24 females [80.0%]). Concerning the DA group, 27 patients were on cabergoline (90.0%) and 20 patients used DA for >2 years (66.7%). The mean current DA dose was 0.62 ± 0.50 mg/week. The surgical group underwent surgery 26 [12-39] months before the cognitive assessment. Twenty-eight (93.3%) surgical patients had received prior DA treatment but were off medication since surgery. At the time of assessment, prolactin levels were 0.4 ± 0.3 the upper limit of normal. The median duration of biochemical control/remission was 31 [13-77] months.

**Table 1.** Characteristics at time of cognitive assessment

		All patients N = 60	All matched healthy controls N = 60	Patients controlled on DA N = 30	Healthy controls (DA) N = 30	Patients in surgical remission N = 30	Healthy controls (surgery) N = 30
**General characteristics**							
Gender (female)		41 (68.3)	41 (68.3)	17 (56.7)	17 (56.7)	24 (80.0)	24 (80.0)
Age*^a,b^* (y)		42.3 ± 11.7	40.6 ± 12.7	45.5 ± 12.6	44.2 ± 13.4	39.2 ± 9.9	37.0 ± 11.1
Education	Low	4 (6.7)	4 (6.7)	0 (0.0)	0 (0.0)	4 (13.3)	4 (13.3)
	Medium	19 (31.7)	19 (31.7)	12 (40.0)	12 (40.0)	7 (23.3)	7 (23.3)
	High	37 (61.7)	37 (61.7)	18 (60.0)	18 (60.0)	19 (63.3)	19 (63.3)
Previous night’s sleep*^a^*	h: min	7:00 ± 1:33	7:23 ± 1:02	6:53 ± 1:22	7:16 ± 1:00	7:07 ± 1:43	7:31 ± 1:03
Hormonal contraceptives*^a^*	Systemic	6 (10.0)	15 (25.0)	2 (6.7)	5 (16.7)	4 (13.3)	10 (33.3)
	Local	6 (10.0)	6 (10.0)	3 (10.0)	2 (6.7)	3 (10.0)	4 (13.3)
Caffeine (units/week)*^a^*		20.6 ± 17.1	20.7 ± 18.0	24.0 ± 19.8	20.0 ± 13.2	16.2 ± 12.8	21.4 ± 15.5
Current smoking*^a^*		8 (13.3)	10 (16.7)	3 (10.0)	5 (16.7)	5 (16.7)	5 (16.7)
Alcohol (units/week)*^a^*	≤2	41 (68.3)	29 (48.3)	20 (66.7)	18 (60.0)	21 (70.0)	11 (36.7)
	3-7	11 (18.3)	23 (38.3)	5 (16.7)	8 (26.7)	6 (20.0)	15 (50.0)
	8-14	6 (10.0)	5 (8.3)	3 (10.0)	2 (6.7)	3 (10.0)	3 (10.0)
	>14	2 (3.3)	3 (5.0)	2 (6.7)	2 (6.7)	0 (0.0)	1 (3.3)
**Disease characteristics**							
Control/remission duration*^c^* (months)		31 [13-77]	-	41 [14-111]	-	29 [13-65]	-
Disease duration*^d^* (months)		60 [28-118]	−	54 [24-126]	−	60 [60-112]	−
Prolactin at diagnosis (×ULN)*^e^*		4.6 [2.8-23.8]	−	15.0 [2.9-29.2]	−	4.0 [2.6-7.2]	−
Current prolactin (×ULN)		0.4 ± 0.3	−	0.4 ± 0.3	−	0.5 ± 0.2	−
Tumor size at diagnosis	Not visible *^f^*	2 (3.3)	−	2 (6.7)	−	0 (0.0)	−
	Micro	30 (50.0)	−	11 (36.7)	−	19 (63.3)	−
	Macro	24 (40.0)	−	14 (46.7)	−	10 (33.3)	−
	Giant	4 (6.7)	−	3 (10.0)	−	1 (3.3)	−
Current tumor size*^g^*	Not visible	30 (51.7)	−	2 (6.7)	−	28 (100.0)	−
	Micro remnant	15 (25.9)	−	15 (50.0)	−	0	−
	Macro remnant	13 (22.4)	−	13 (43.3)	−	0	−
	Giant remnant	0	−	0	−	0	−
Current hypopituitarism*^h^*	Any axis	12 (20.0)	−	8 (26.7)	−	4 (13.3)	−
	LH/FSH	8 (13.3)	−	7 (23.3)	−	1 (3.3)	−
	TSH	4 (6.7)	−	2 (6.7)	−	2 (6.7)	−
	ACTH	2 (3.3)	−	1 (3.3)	−	1 (3.3)	−
	GH	1 (1.7)	−	0	−	1 (3.3)	−
	AVP	1 (1.7)	−	0	−	1 (3.3)	−
**Treatment**							
Current (DA)	Yes	30 (50.0)	−	30 (100.0)	−	0	−
	Cabergoline	27 (90.0)	−	27 (90.0)	−	0	−
	Quinagolide	1 (3.3)	−	1 (3.3)	−	0	−
	Bromocriptine	2 (6.7)	−	2 (6.7)	−	0	−
Previous	DA	58 (96.7)	−	30 (100.0)	−	28 (93.3)	−
	TSS*^i^*	32 (53.3)	−	2 (6.7)	−	30 (100.0)	−
	Radiotherapy	0	−	0	−	0	−
Duration DA treatment	< 6 m	6 (10.3)	−	1 (3.3)	−	5 (17.9)	−
	6 m-2 y	15 (25.9)	−	8 (26.7)	−	7 (25.0)	−
	> 2 y	36 (62.1)	−	20 (66.7)	−	16 (57.1)	−
	Unknown	1 (1.7)	−	1 (3.3)	−	0	−
Current DA dose *^j^* (mg/week)		0.62 ± 0.50	−	0.62 ± 0.50	−	0	−
Time since surgery (months)		25 [12-39]	—	31 [22-31]	—	26 [12-39]	—

Data reported as value (%), mean ± SD, or median [interquartile range].

Abbreviations: AVP, arginine vasopressin; DA, dopamine agonist; NA, not applicable; TSS, transsphenoidal surgery; ×ULN times upper limit of normal (females 23.3 µg/L, males 15.2 µg/L).

^*a*^Characteristics were statistically compared between (1) all patients and (2) all controls, patients controlled on DA and their matched healthy controls, and (3) patients in surgical remission and their matched healthy controls. No significant differences were found (*P* > .050).

^*b*^Statistical testing was performed despite of matching, as 10 years age difference was accepted.

^*c*^Defined as time from achievement of sustained normoprolactinemia to cognitive assessment, available for n = 55 (patients controlled on DA: n = 28, patients in surgical remission: n = 27).

^*d*^Defined as time from diagnosis to the cognitive assessment.

^*e*^Data available for 54 patients (patients controlled on DA: n = 26, patients in surgical remission: n = 28).

^*f*^Both patients developed a visible adenoma throughout the disease course.

^*g*^Data available for 58 patients; postoperative magnetic resonance imaging was not yet performed in 2 surgically treated patients.

^*h*^All patients were substituted adequately according to international guidelines, except for 1 asymptomatic male with mild hypogonadism in the DA group who refused testosterone substitution.

^*i*^Two patients in surgical remission had undergone 2 surgeries.

^*j*^In equivalents of cabergoline: bromocriptine 2.5 mg/day or quinagolide 75 µg/day were considered to be the equivalent of cabergoline 0.5 mg/week.

Hypopituitarism of any axis was present in 12 (20.0%) patients, concerning the gonadotropic axis in 8 patients (13.3%). Hypopituitarism was substituted adequately in all but 1 asymptomatic, mildly hypogonadotropic male refusing testosterone treatment.

None of the 30 surgically treated patients had adenoma remnants on most recent MRI (missing data, n = 2, 6.7%). Concerning DA-treated patients, a microadenoma remnant was present in 15 patients (50.0%), a macroadenoma remnant in 13 patients (43.3%), and no remnant was visible in 2 patients (6.7%).

The self-reported disease burden as measured by LBNQ-Pituitary, indicated the highest burden in the Physical and Cognitive Complaints, followed by the Mood Symptoms domain for both DA-treated and surgically treated patients. An overview of LBNQ-Pituitary scores is presented in Table 2. Concerning individual questions, 24 (40.0%) patients indicated mood disturbances because of their pituitary disease. Memory and concentration problems were indicated by 37 (61.7%) and 28 (46.7%) patients, respectively.

**Table 2.** Self-reported disease burden for patients, as measured by LBNQ-Pituitary

LBNQ-Pituitary	All patients N = 60	Patients controlled on DA N = 30	Patients in surgical remission N = 30
**Bothers**			
Mood problems	6.9 [0.0-29.9]	12.5 [0.0-29.9]	5.6 [0.0-31.3]
Negative illness perception	2.1 [0.0-12.5]	4.2 [0.0-13.5]	0.0 [0.0-9.4]
Issues in sexual functioning	0.0 [0.0-12.5]	0.0 [0.0-18.8]	0.0 [0.0-14.1]
Physical and cognitive complaints	16.7 [2.8-38.9]	18.1 [2.8-38.9]	15.3 [0.0-22.4]
Issues in social functioning	0.0 [0.0-10.0]	0.0 [0.0-12.5]	0.0 [0.0-10.0]
Total	8.7 [1.1-22.7]	8.3 [2.5-23.5]	9.1 [0.8-22.2]
**Needs**			
Mood problems	8.3 [0.0-25.0]	8.3 [0.0-22.9]	6.9 [0.0-31.9]
Negative illness perception	0.0 [0.0-15.6]	2.1 [0.0-16.7]	0.0 [0.0-13.5]
Issues in sexual functioning	0.0 [0.0-12.5]	0.0 [0.0-14.1]	0.0 [0.0-12.5]
Physical and cognitive complaints	11.1 [0.7-36.1]	11.1 [2.1-38.2]	12.5 [0.0-37.5]
Issues in social functioning	0.0 [0.0-7.5]	0.0 [0.0-11.3]	0.0 [0.0-2.5]
Total	8.0 [1.0-22.5]	8.0 [1.3-24.2]	7.6 [0.8-23.7]

Data reported as median [interquartile range].

Abbreviation: LBNQ-Pituitary Leiden Bothers and Needs Pituitary.

### Full Cohort

#### Cognitive functioning

**Figure 2.**
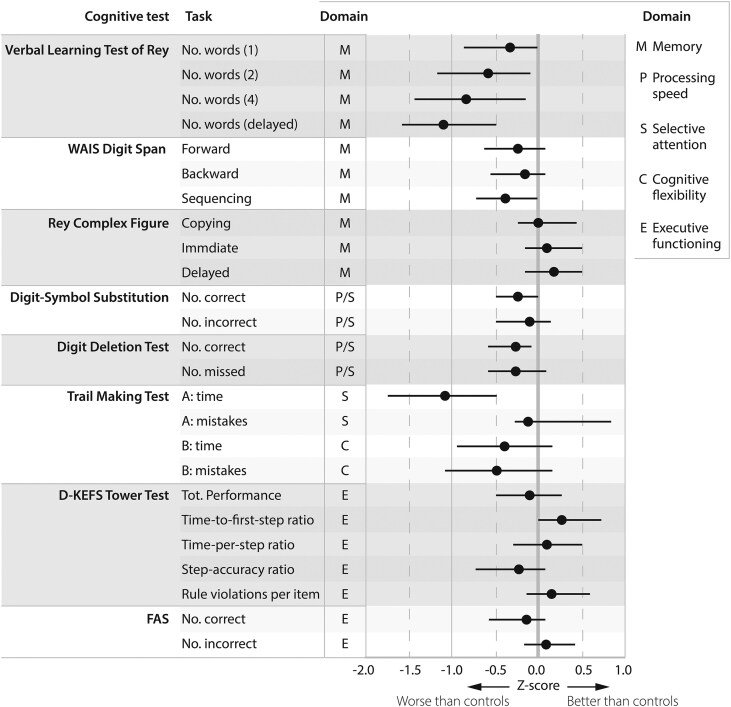
Results of the cognitive assessment for biochemically controlled patients after medical treatment or surgery (n = 60). The cognitive domain tested is indicated per test. Data are displayed as *Z*-scores for patients based on matched healthy controls, and reported as mean with 95% CIs. Lower *Z*-scores indicate patients scoring worse than controls, and vice versa. For the Verbal Learning Test of Rey the first, second, fourth, and delayed attempt are shown because these provide the most relevant information. Abbreviations: No., number; tot., total.

The outcomes of the cognitive assessment are summarized in Fig. 2 and Supplementary Table S3 (13, 14). Using *Z*-scores for the overall comparison of patients to controls, patients scored 0.2 to 1.1 SD lower than controls on tasks measuring verbal memory (Rey Complex Figure Test, Wechsler Adult Intelligence Scale [WAIS] Digit Span Task). Patients performed significantly worse on all attempts of the Verbal Learning Test of Rey (fewer reproduced words - most pronounced on the delayed attempt: β = −1.8 (95% CI, −2.7 to −1.0), *P* < .001). Patients performed −0.2 to −1.1 SD worse on tasks assessing attention, with fewer correct substitutions on WAIS Digit-Symbol Substitution Test (β = −4.2 [95% CI, −8.2 to −0.2], *P* = .040), and more time (β = 5.2 [95% CI, 3.2 to 7.2], *P* < .001) and a higher number of mistakes on TMT-A (β = .1 [95% CI, 0.0 to 0.2], *P* = .028). Patients performed −0.2 to −0.4 SD worse on tasks assessing processing speed (digit deletion test: fewer correctly deleted digits (β = −8.8 [95% CI, −16.2 to −1.4], *P* = .019), and WAIS Digit-Symbol Substitution Test). Patients performed similar to matched controls on tasks assessing visuospatial memory (Rey Complex Figure Test), cognitive flexibility (TMT-B), and executive functioning (TT, FAS).

#### Psychological complaints and personality traits

**Figure 3.**
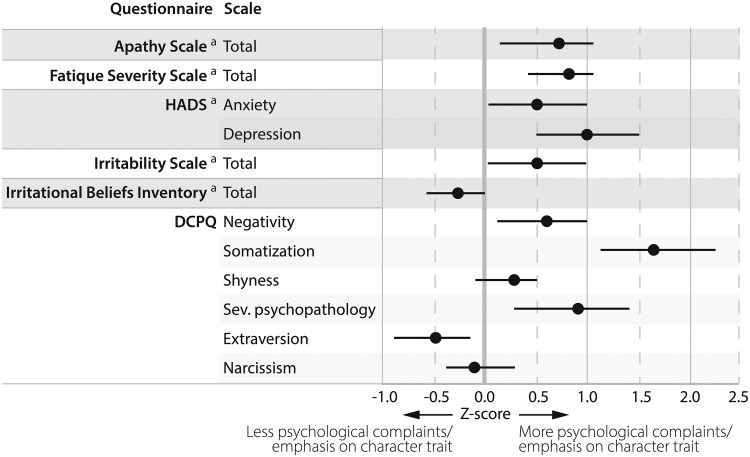
Results of patient-reported outcome measures concerning psychological complaints and maladaptive personality traits for biochemically controlled patients after medical treatment or surgery (n = 60). Data are displayed as *Z*-scores for patients based on matched healthy controls, and reported as mean with 95% CIs. Lower *Z*-scores indicate patients reporting less psychological complaints or emphasis on a character trait than controls, and vice versa. ^a^Data missing for 1 surgically treated patient who did not complete all patient-reported outcome measures (n = 59). Abbreviations: DCPQ, Dutch Clinical Personality Questionnaire; HADS, Hospital Anxiety and Depression Score; sev., severe.

As shown in Fig. 3 and Supplementary Table S4 (13, 14), patients generally reported more psychological symptoms than controls. Patients reported significantly more apathy (AS: β = 2.4 [95% CI, 0.6 to 4.1], *P* = .009), more fatigue (*Fatigue Severity Scale*: β = 6.7 [95% CI, 2.7 to 10.8], *P* < .001), more irritability (IPS: β = 2.2 [95% CI 0.3 to 4.1], *P* = .024), more anxiety (HADS anxiety score: β = 1.1 [95% CI, 0.1 to 2.1], *P* = .034), and more depressive symptoms (HADS depression score: β = 1.7 [95% CI. 0.8 to 2.7], *P* < .001). Using the HADS, clinically relevant symptoms of depression were observed in 5 (8.3%) patients, and anxiety in 15 (25.4%) patients, respectively. There were no significant differences regarding irrational beliefs (Irrational Beliefs Inventory-50) between patients and controls.

Concerning maladaptive personality traits (DCPQ), patients exhibited higher levels of negativism (β = 2.8 [95% CI, 0.8 to 4.9], *P* = .007), somatization (β = 7.3 [95% CI, 5.0 to 9.5], *P* < .001), severe psychopathology (β = 1.9 [95% CI, 0.8 to 3.1], *P* = .001), and lower levels of extraversion (β = −3.7 [95% CI, −6.7 to −0.7], *P* = .015) compared to controls. Degrees of shyness and narcissism were comparable between patients and controls.

### Comparison of Medically and Surgically Treated Patients

#### Cognitive functioning

**Figure 4.**
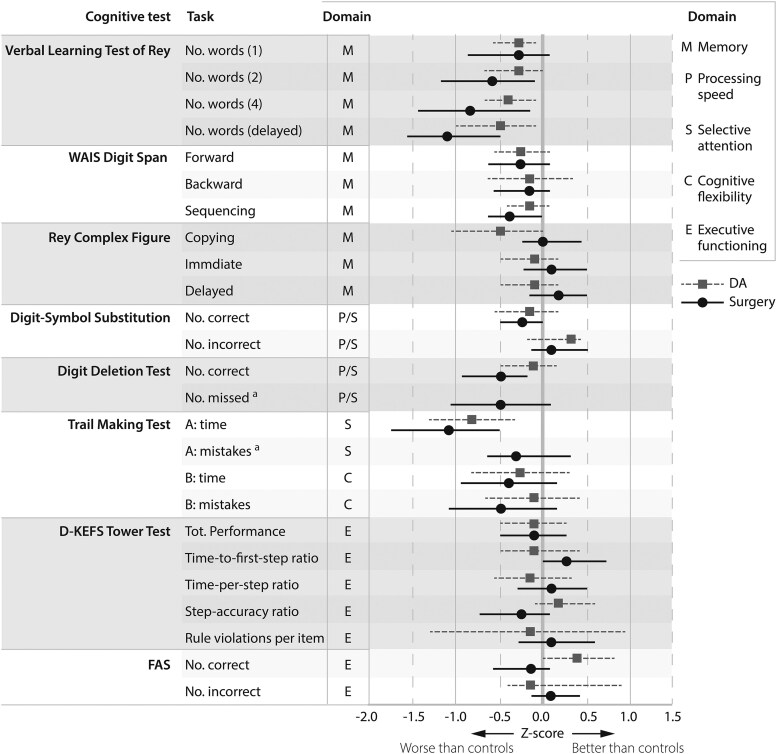
Results of the cognitive assessment for patients controlled on DA (n = 30) and patients in surgical remission (n = 30). Data are displayed as *Z*-scores for patients based on matched healthy controls, and reported as mean with 95% CIs. Lower *Z*-scores indicate patients scoring worse than controls, and vice versa. Differences in disease characteristics may exist between patients controlled on DA and patients in surgical remission as they were not matched. For the Verbal Learning Test of Rey the first, second, fourth, and delayed attempt are shown, as these provide the most relevant information. ^a^*Z*-score could not be calculated for patients controlled on DA as the mean and standard deviation were zero for the matched controls. Abbreviations: DA, dopamine agonist; no., number; tot., total.

Baseline characteristics and results of the LBNQ-Pituitary for the treatment groups are shown in Tables 1 and 2, respectively. None of the patient groups had mean *Z*-scores >2.0 SD below the control group's mean on any outcome of the cognitive assessment. Surgically and DA-treated patients generally showed comparable results on the cognitive tests, as depicted in Fig. 4. Differences were marginal and distributed randomly across cognitive domains, thus most likely resulting from multiple testing. An overview of absolute test results for the patient groups and controls, and *Z*-scores of the outcomes of the cognitive assessment are provided in Supplementary Tables S5 and 6, respectively (13, 14).

#### Psychological complaints and maladaptive personality traits

**Figure 5.**
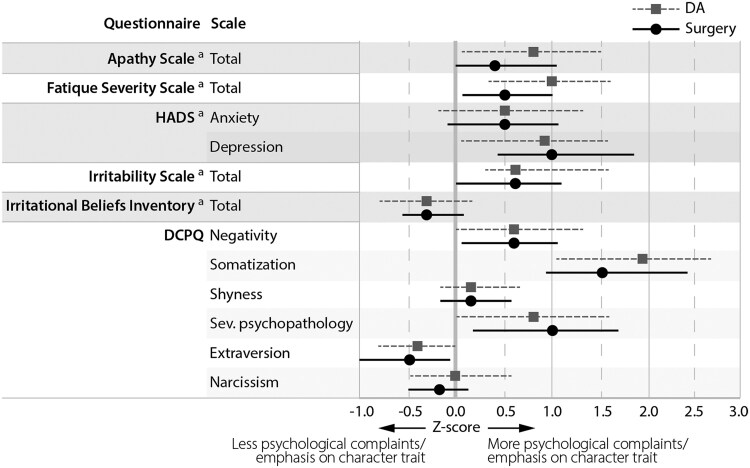
Results of patient-reported outcome measures concerning psychological complaints and maladaptive personality traits for patients controlled on DA (n = 30) and patients in surgical remission (n = 30). Data are displayed as *Z*-scores for patients based on matched healthy controls, and reported as mean with 95% CIs. Lower *Z*-scores indicate patients reporting less psychological complaints or emphasis on a character trait than controls, and vice versa. Differences in disease characteristics may exist between patients controlled on DA and patients in surgical remission as they were not matched. ^a^Data missing for 1 surgically treated patient who did not complete all patient-reported outcome measures. Abbreviations: DCPQ, Dutch Clinical Personality Questionnaire; HADS, Hospital Anxiety and Depression Score; sev., severe.

None of the patient groups had mean *Z*-scores >2.0 SD below the control group's mean on any PROM. Surgically and DA-treated patients scored comparably on all PROMs. Concerning maladaptive personality traits, both patient groups scored highest on somatization, as depicted in Fig. 5. An overview of absolute results for the patient groups and controls, and *Z*-scores for PROMs are provided in Supplementary Tables S7 and 8, respectively (13, 14).

### Factors of Influence for Cognitive Functioning

*Z*-scores of cognitive tests were not correlated to prolactin at diagnosis, duration of biochemical control/remission, presence of pituitary deficiencies, or total HADS scores. Outcomes of multilinear regression analyses are shown in Supplementary Table S9 (13, 14).

## Discussion

Clinical experience suggested that cognitive and psychological complaints are prevalent among patients with prolactinoma and can persist after disease control. This study was the first to compare a large group of patients with biochemically controlled prolactinoma to matched controls, enabling additional comparison of treatment modalities using *Z*-scores. Patients with diagnosed psychological comorbidity were excluded to avoid bias, potentially leading to underestimation of cognitive impairments and psychological complaints. Even in this selected group, our clinical observations were confirmed with 40% of the patients self-reporting mood disturbances, and half of the patients self-reporting memory and concentration problems. This study demonstrated subtle cognitive impairments, and more psychological complaints and maladaptive personality traits in biochemically controlled patients compared to controls.

Previous research in untreated patients with prolactinoma demonstrated cognitive impairments, including impairments in verbal memory, working memory, attention, and executive functioning (33-36). An overview of available studies on cognitive functioning in prolactinoma cohorts is provided in Table 3. Two studies prospectively examining cognitive functioning showed improvement of response activation (using electroencephalogram measurements), and processing speed, working memory, visual learning and reasoning, and problem solving after treatment (6, 7). One cross-sectional study found no differences in reaction times and accuracy between surgically treated patients and controls (5). The results of our cross-sectional study in patients with biochemically controlled prolactinoma showed subtle cognitive impairments (ie, memory, attention, processing speed), suggesting that these impairments may persist after biochemical normalization. We hypothesized cognitive functioning would be impaired to a greater extent in DA-controlled patients compared to patients in surgical remission because DAs may induce (cognitive) side effects. However, after correction for age, gender, and education level, DA-controlled patients and patients in surgical remission scored similarly on the cognitive tasks. This lack of a significant difference might be explained by good tolerance to DA in the medically treated patients because DA side effects were an indication for neurosurgical intervention.

**Table 3.** Overview of studies reporting on cognitive functioning in patients with prolactinoma

Author (year)	Subjects	Controls	Design	Outcomes
Psaras (6) (2011)	Patients: N = 106 patients with a pituitary adenoma (female: 69 [65%]), among which 12 patients with a prolactinoma (female: NR) Exclusion: Craniopharyngioma, metastasis, psychiatric or neurological disorders, major comorbidity known to affect neurocognitive functions (kidney or liver disease), insufficient hearing ability, uncorrected visual abnormalities	None	Longitudinal study: Measurement before and 3- and 12-months postsurgery Assessment: D2 Letter Cancellation test (selective attention), WAIS Digit Span Task (working memory), Trail-Making-test A (attentional speed), Intelligence Structure Test-2000 (episodic memory)	All: Improvement of concentration, working memory, and attentional speed within 3 months. Improvement of episodic memory after 12 months. Prolactinoma: Removal of suprasellar extension was the most important factor for improvement of neurocognitive functions.
Montalvo (7) (2018)	Patients: Microprolactinoma N = 7 (female: 6 (86%)) Exclusion: Mental illness, prolactin-elevating drugs, substance use, mental retardation, dementia, other hyperprolactinemia causing diseases, pregnancy	None	Longitudinal study: Measurement before and 6-12 months after starting cabergoline Assessment: Brief Assessment of Cognition in Schizophrenia-Symbol Coding (speed of processing), Category Fluency-Animal Naming test (speed of processing), Trail Making Test Part A (selective attention), Continuous Performance Test-Identical Pairs (attention and vigilance), WMS-III Spatial Span (working memory), University of Maryland Letter-Number Span (working memory), Hopkins Verbal Learning Test-Revised (verbal learning), Brief Visuospatial Memory Test-Revised (visual learning) and Neuropsychological Assessment Battery-Mazes (reasoning and problem solving)	Improvement of processing speed, working memory, visual learning, reasoning and problem-solving after starting cabergoline.
Yao (35) (2018)	Patients: Currently untreated prolactinoma, with hyperprolactinemia, ≥ 9 years of Education N = 32 (female: 32 (100%)) Exclusion: Left-handed, in pubertal stage, significant VFD, history of neurological or psychiatric disorders, acquired brain injury, substance abuse, smoking >10 cigarettes/day, medication use (including DA and oral contraceptives)	Healthy volunteers matched for age, sex, education, and handedness N = 26	Cross-sectional study Assessment: Structural MRI Wisconsin Card Sorting Test (executive functioning), Picture Recall Test, Visual Recognition Test and Story Recall tests (all assessing nonverbal or verbal memory)	Patients scored worse on verbal memory and executive functioning. Furthermore, patients showed a decreased grey matter volume of the left hippocampus, left orbitofrontal cortex, right middle frontal cortex and right interior frontal cortex. Impairments of verbal memory and executive functioning were associated with gray matter volume of the left hippocampus and right middle frontal cortex.
Bala (32) (2022)	Patients: Treatment-naïve prolactinoma N = 27 (female: 15 [56%]) Exclusion: History of neurologic or psychiatric disorders, use of medication (including DA, oral contraceptives), pregnancy, substance abuse, significant VFD	Healthy, not pregnant, no medication. Matched based on sex, age, education, handedness, mood level N = 27	Cross-sectional study Assessment: D2 Test of Attention and Color Trails Test (selective attention and concentration), Color Trails Test (spatial screening and working memory), Elevator Counting (sustained attention), Visual Elevator (attentional switching), Telephone Search and Telephone Search while Counting (visual screening and divided attention), Digit Span (auditory-verbal working memory), Symbol Span (spatial working memory)	Patients scored worse on selective attention, spatial screening and spatial working memory, auditory-verbal working memory, attentional switching, visual screening and divided attention. Higher prolactin levels were associated with lower scores in some working memory and attention assessments.
EEG studies				
Cao (34) (2020)	Patients: Currently untreated prolactinoma, 20-50 years old, > 9 - ≤ 15 years of education. N = 21 (female: 14 [67%]) Exclusion: Radiotherapy, craniotomy, history of neurologic or psychiatric disorders, comorbidities that could impair cognitive function (severe kidney, liver or heart disease), coma, infections, epilepsy, hydrocephalus, CSF leakage, substance abuse, medication use (including oral contraceptives) insufficient hearing ability, uncorrected visual abnormalities	Healthy volunteers matched for age and education level N = 21	Cross-sectional study Assessment: EEG, Visual Go/Nogo task	Impaired response activation and inhibition with lower frontal theta activity and occipital alpha activity in both Go and Nogo conditions in PRL compared to healthy controls. Mediator model suggested that the relationship between frontal theta power and inhibitory ability was mediated by increased prolactin levels. Negative relationship between prolactin levels and gray matter volume of left hippocampus and right inferior frontal cortex.
Song (5) (2020)	Patients: Prolactinoma resistant or intolerant to long-term drug treatment Presurgery: N = 20 (female: 10 [50%]) Six months postsurgery: N = 20 (female: 10 (50%)) Exclusion: History of craniotomy or radiotherapy, neurologic or psychiatric disorders, comorbidity that can affect cognitive function (kidney, liver, or heart disease), severe complications (eg, hydrocephalus, CSF leak), substance abuse, medication use (including oral contraceptives)	Healthy volunteers matched for age, gender, and education N = 20	Cross-sectional study Assessment: EEG, Visual Go/Nogo task	Longer reaction times and lower accuracy in active PRL compared to post-surgical PRL and healthy controls. Generally, no difference between reaction times and accuracy in postsurgical PRL compared to healthy controls. No correlation between prolactin levels and amplitude of P300 in both Go and Nogo conditions.
Cao (33) (2023)	Patients: Currently untreated prolactinoma resistant to long-term medical therapy N = 26 (female: 18 (69%) Exclusion: Radiotherapy, craniotomy, history of neurologic or psychiatric disorders, comorbidities that could impair cognitive function (severe kidney, liver, or heart disease), coma, infections, epilepsy, hydrocephalus, CSF leakage, substance abuse, medication use (including DA and oral contraceptives) insufficient hearing ability, uncorrected visual abnormalities	Healthy volunteers matched for age and education level N = 26	Cross-sectional study Assessment: EEG, Color-shape Switching Task (task switching)	Patients showed longer reaction time in switch trials and larger switch costs. Weaker frontal theta activity and disrupted frontoparietal connectivity in patients compared to healthy controls. Higher prolactin levels were associated with larger decrease in cognitive performance.

Data are presented as value (%) or mean ± SD, unless stated otherwise.

Abbreviations: CSF, cerebrospinal fluid; DA, dopamine agonist; EEG, electroencephalogram; NR, not reported; TSS, transsphenoidal surgery; VFD, visual field defects.

Although comparison of cognitive impairments between pituitary diseases is difficult due to differences in cognitive assessment protocols, similar impairments in verbal learning, attention, and processing speed were found in patients with long-term (on average 13 years) remission of Cushing disease (CD) (37). By contrast, no cognitive impairments were found in patients after long-term remission of acromegaly or nonfunctioning pituitary adenoma (NFPA) (38). As the present study was cross-sectional, and relatively short-term after intervention, long-term cognitive outcomes in patients with treated prolactinoma remain unknown. The observed impairments may still be reversible (with appropriate rehabilitation). Therefore, monitoring cognitive complaints and referring to cognitive rehabilitation facilities is important for this group, in whom these problems are less acknowledged than in CD. Monitoring cognitive complaints can be performed during routine clinical visits by using (open-ended) screening questions (eg, “how is memory going?”, “how is concentrating, such as in following the plot of a movie or book?”, Do you need more time to process information and/or solve problems?”) potentially supported by using validated questionnaires (eg, cognitive failure questionnaire, LBNQ-Pituitary). Furthermore, the opinion of a patient's partner/spouse on these issues should be included when available.

Concerning psychological complaints, higher degrees of apathy, fatigue, irritability, anxiety, and depression were reported by patients compared to controls, which was in line with a recent systematic review on health-related quality of life, indicating the most pronounced impairments in the mental health domain (4): more fatigue, poorer sleep quality, and shorter sleep duration (2, 39, 40). Albeit equivocal, symptoms generally improved—without normalization—after biochemical control (3, 4, 9, 41, 42). Our group previously studied psychological complaints and personality traits using the same PROMs in patients with acromegaly (n = 68), CD (n = 51), and NFPA (n = 60) in long-term biochemical control (37, 38, 43), enabling comparison of *Z*-scores. Psychological complaints in patients with biochemically controlled prolactinoma were similar to complaints in CD, acromegaly, and NFPA. Patients with treated prolactinoma, acromegaly, and NFPA scored 0.5 to 1.0SD worse than matched controls, whereas patients with CD scored 1.0 to 2.0 SD worse than matched controls (38, 43). Furthermore, these previous studies demonstrated more maladaptive personality traits (ie, negative affect, lack of positive affect, somatic arousal) in treated patients with CD and acromegaly compared to controls (37, 43). Athanasoulia et al observed more neuroticism, increased sensitivity to negative emotional stress, increased fear of uncertainty, and higher degrees of socially desirable behavior in patients with prolactinoma (controlled on DA and active) compared to healthy controls (38). Thus, previous and present findings suggest that psychological complaints are prevalent in patients with pituitary adenoma after biochemical normalization—irrespective of the (type of) hormonal hypersecretion. Therefore, these psychological complaints, and personality traits, should be addressed in both patients with active disease, and after biochemical control. Monitoring these psychological complaints can be supported by validated PROMs (eg, LBNQ-Pituitary, HADS), and patients should be referred to a psychologist/social worker and/or self-management programs upon indication (44).

The underlying mechanisms of these subtle cognitive impairments and psychological complaints may be multifactorial. First, hyperprolactinemia might induce (ir)reversible alterations in the brain. Accordingly, alterations in brain activity and brain structures have been found in patients with active prolactinoma (Table 3) (5, 34-36) and these structural alterations (ie, decreased gray matter volume of the left hippocampus, left orbitofrontal cortex, right middle frontal cortex, and right interior frontal cortex) were negatively correlated with verbal memory and executive functioning (36). Additionally, prolactin levels and cognitive impairments were positively associated in some (33-35), yet not all studies (5). In our cohort, prolactin levels at diagnosis did not correlate with the cognitive assessment after treatment, potentially because of (partial) reversibility, variable sensitivity to the effects of prolactin, or a lack of power. Second, dopamine—an important neuroregulator of working memory and cognitive control, among others—may impact cognitive function (45). In patients with prolactinoma the dopaminergic tone may be altered bidirectionally, with hyperprolactinemia suppressing dopamine due to dopaminergic neurons becoming refractory, and medical treatment increasing dopaminergic tone (46). The current use of DAs did not clearly affect cognitive functioning in our cohort, as similar outcomes were observed in DA- and surgically treated patients. However, ongoing effects of prior DA treatment in the surgically treated patients cannot be excluded. Furthermore, surgically and medically treated patients differed in some disease characteristics, which could not completely be accounted for statistically: surgically treated patients were generally DA intolerant, and the DA-treated patients may have had irresectable/larger tumors. The role of hypopituitarism remains unclear. One study in patients with CD in remission reported that hypopituitarism was associated with more impairments in cognitive functioning (43). By contrast, the present and previous studies observed no correlation between treated hypopituitarism and cognitive impairments, with previous studies indicating mild-to-no objectifiable deficits in treated primary and secondary adrenal insufficiency (47), and unconvincing effects of sex hormone replacement on cognitive functioning in (mostly elderly) individuals (48, 49). Last, it is well known that mood disorders can influence cognitive functioning (50). However, this association was not observed in the present study, potentially because of exclusion of patients with diagnosed psychopathology. Taken together, underlying mechanisms of cognitive impairments require further analysis.

Multiplicity is inevitable when assessing cognition and psychopathology because it involves assessment of multiple domains using separate testing instruments. Multiple testing corrections were not appropriate because of underlying association of the endpoints. Despite this being the largest prolactinoma population assessed for cognition and psychological complaints to date, the power to detect subtle associations might be lacking. Nevertheless, clear trends were observed in patients with biochemically controlled prolactinoma.

The current study is a starting point for further, in-depth exploration of cognitive functioning. Future studies should include patients who underwent primary surgical treatment without DA pretreatment to examine DA effects, and longitudinal pre- and posttreatment testing to assess the degree of reversibility of cognitive impairments and psychological complaints in both surgically and medically treated patients. Additionally, functional, and structural brain MRI studies can provide insight into the course of (potentially persisting) cerebral alterations. Moreover, patients with prolactinoma should be compared to patients with other chronic (hormonal) conditions to elucidate prolactin- or dopamine-specific effects on the brain. Last, the added value of screening tools and cognitive rehabilitation programs should be formally evaluated.

In conclusion, patients with biochemically controlled prolactinoma demonstrated subtle cognitive impairments, pertaining to verbal memory, attention, and processing speed, compared to controls. Furthermore, patients reported more psychological complaints and maladaptive personality traits compared to controls. Physicians should be aware of these impairments and complaints and address these issues appropriately. Further research should encompass longitudinal assessments and the evaluation of the potential added value of cognitive rehabilitation and psychological support programs.

## Data Availability

The dataset analyzed during the current study is not publicly available but is available from the corresponding author on reasonable request.

## Abbreviations

CD: Cushing disease
DA: dopamine agonist
DCPQ: Dutch Clinical Personality Questionnaire
HADS: Hospital Anxiety and Depression Scale
LBNQ: Leiden Bothers and Needs Pituitary
MRI: magnetic resonance imaging
NFPA: nonfunctioning pituitary adenoma
PROM: patient-reported outcomes measures
TMT: Trail Making Test
TSS: transsphenoidal surgery
